# The potential of cancer stem cells for personalized risk assessment and therapeutic intervention in individuals with intrahepatic cholangiocarcinoma

**DOI:** 10.1007/s12672-024-01179-7

**Published:** 2024-07-24

**Authors:** Jian Zhang, Tao Cui, Jiaobang Xu, Peng Wang, Chongqing Lv, Guozheng Pan

**Affiliations:** Hepatobiliary Surgery, Shengli Oilfield Central Hospital, Dongying, 257093 China

**Keywords:** Intrahepatic cholangiocarcinoma, Cancer stem cells, Prognosis, Chemotherapy drugs, Immunotherapy

## Abstract

**Background:**

Accumulating evidence suggests that intrahepatic cholangiocarcinoma (ICC) is a stem cell-based disease, but information on the biology of cancer stem cells (CSC) in ICC is very limited.

**Methods:**

ICC RNA-seq cohorts from three different public databases were integrated and the protein-coding genes were divided into different modules using "WGCNA" to screen the most relevant modules with CSC scores. Least Absolute Shrinkage and Selection Operator (LASSO) regression were introduced to construct prognostic classification models. In addition, the extent of immune cell infiltration in patients in different risk groups was assessed based on the ESTIMATE, CIBERSORT, MCP-Counter, and single sample gene set enrichment analysis (ssGSEA) algorithms. Finally, the correlation between different risk scores and common drugs was analyzed by pRRophetic package and Spearman method.

**Results:**

In the present study, we found that a high CSC score was associated with a poorer prognosis in patients with ICC. The yellow module obtained by WGCNA was significantly positively correlated with the CSCs score, in which 8 genes were served to build a prognostic classification model, and the obtained risk score was negatively correlated with CSCs score and prognosis. The low-risk score was more suitable for immunotherapy, and the high-risk score was more suitable for treatment with 11 antitumor drugs.

**Conclusion:**

This study revealed the regulatory role of CSC-mediated EMT, angiogenesis, and immunomodulatory biological processes in ICC, and applied a prognostic classification model to highlight the great potential of CSC for personalized risk assessment, chemotherapy, and immunotherapy intervention in ICC individuals.

**Supplementary Information:**

The online version contains supplementary material available at 10.1007/s12672-024-01179-7.

## Introduction

Intrahepatic cholangiocarcinoma (ICC) is the second most common primary liver tumor, accounting for 20% of all liver malignant tumors and 3% of all gastrointestinal malignancies [1, 2]. Surgical resection remains the only curative option; however, only 20%–30% of patients are eligible for resection [3, 4]. It is reported that five years after resection relapse-free survival rates of 10% to 31% [5, 6]. Systemic therapy is the sole mainstay for 70 to 80% of patients with locally unresectable or distant metastatic disease, but survival remains limited to about 1 year [1]. In recent decades, there has been a gradual rise in the occurrence of ICC worldwide, particularly among individuals with preexisting liver conditions [7, 8]. For example, Yao et al. constructed prognostic models and biomarkers with good predictive value based on ferroptosis-related genes [9]. However, there is still a lack of comprehensive understanding of the biogenesis associated with ICC, which has hindered progress in the treatment of patients with ICC. Hence, it is critical to investigate fresh prognostic markers and uncover possible mechanisms to enhance comprehension of disease advancement.

Cancer stem cells (CSCs) are a small subpopulation of cancers with long-term in vivo tumorigenicity, which have been proposed as cancer initiating cells, and shown to be the cause of chemotherapy resistance and cancer recurrence [10–12]. In a typical liver, the intrahepatic biliary epithelium may proliferate in a liver injury model, but the proliferation of biliary epithelial cells relies on a randomly sustained progenitor population [13]. In the case of cholangiocarcinoma, the existence of CSCs has been theorized in pathological observations conducted many years in the past. In rats fed a diet lacking choline and acetylaminofluorene, oval cells (which are liver stem cells) proliferated, leading to the occurrence of cholangiocarcinoma [14]. Furthermore, the transcriptomic analysis of patient tissues indicated that the gene expression patterns in iCC overlapped with those of hepatocellular carcinoma and displayed features of stem cell gene expression [15]. In recent years, CSC molecular markers have come into the limelight, which are considered to be important for changing the clinical outcome of patients with this disease [16]. The study of Padthaisong et al. provided four candidate CSC markers CD44, CD44v6, CD44v8-10 and EpCAM with good performance in the prognosis and postoperative recurrence of cholangiocarcinoma [17] CSC markers identified as early diagnosis or prognosis of ICC also include DCLK1 [18], CD133 and CD24 [16]. However, most CSC markers are not selected on the basis of a deep understanding of the underlying stem-cell biology of the tissues in which the cancer originates [19].

In this study, we aimed to explore the prognostic value of CSC-associated genes and to establish a new ICC prognostic model based on CSC-associated genes. We first obtained expression profiles and clinical information of ICC samples based on public databases, as well as stem cell signatures. Subsequently, the weighted gene co-expression network analysis (WGCNA) and Lasso regression analyses were utilized to censor CSCs-related genes and construct risk models. Finally, we evaluated the level of immune cell infiltration and the potential response to immunotherapy in patients at different risks. These findings may provide new prognostic biomarkers for ICC and provide a broader perspective for individualized treatment of patients.

## Material and methods

### Data gathering and preprocessing

RNA-seq cohorts of cholangiocarcinoma tissues from 3 different public databases: TCGA-CHOL (Cholangiocarcinoma) cohort from The Cancer Genome Atlas database (TCGA, https://portal.gdc.cancer.gov) and GSE107943 data set from Gene Expression Omnibus database (GEO, http://www.ncbi.nlm.nih.gov/geo), and E-MTAB-6389 cohort of EBI site (https://www.ebi.ac.uk/arrayexpress/experiments/E-MTAB-6389/). Raw data for each cohort were processed: Only the “intrahepatic Cholangiocarcinoma” samples from the TCGA-CHOL cohort with documented clinical survival data were retained, Ensembl was converted to Gene symbol, and all expression profiles were converted to log2 form. A total of 30 eligible tumor samples were included in the TCGA-CHOL cohort. In the GSE107943 dataset, probes were mapped to genes based on annotation information, and the expression value of genes matched by more than one probe was the mean of the probes. ICC data from three different sources were merged into a single set and batch effects were removed using the remove “BatchEffect” function of the “limma” package [20].

### Calculation of CSC score

StemChecker web server is based on the most extensive and up-to-date curation of published stemness signatures [21]. A total of 155 stemness genes from KEGG and REACTOME were obtained from StemChecker, and the CSCs scores of the samples were calculated using the R package “GSVA” [22], and then the grouping threshold of CSCs scores was delineated using the “survminer” package [23].

### Weighted gene co-expression network analysis (WGCNA)

WGCNA implements a soft threshold procedure using a power function, which is widely used to cluster transcriptome datasets [24]. Clustering of ICC data was achieved using the R package “WGCNA”. The top 50% of protein-coding genes with the largest change in standard deviation of expression were retained. The “pickSoftThreshold” function selected the soft threshold based on the near-scale-free topological criterion. The one-step method was employed to construct the weighted co-expression network with minModuleSize = 50 and other default parameters. Modules obtained by WGCNA were correlated with CSC scores and plotted as a heatmap of module-feature relationships with correlation coefficient and p-value.

### Gene set enrichment analysis (GSEA)

“GSEA” [25] was adopted to analyze the effect of pathway perturbation in the hallmark gene set on the CSCs phenotype of the samples. The enrichment score (ES) of the gene set was calculated, and the statistical significance of ES was estimated using an empirical phenotyping based permutation test procedure. The ES of each gene set was then normalized to produce a normalized enrichment score (NES).

### Construction of prognostic classification model

From the modules identified by WGCNA, the module with the highest correlation with CSCs was used for stepwise gene screening. Univariate Cox regression analysis implemented by the “survival” package [26] were the first to screen the genes in the module with the criteria of p < 0.05. LASSO model was established for the genes screened by univariate Cox regression analysis by introducing the R package “glmnet” [27]. The last filtering step was stepwise multivariate Cox regression analysis. The expression of the genes screened in the previous step in each sample was formed into a data frame with the survival status and survival time of each sample, which was used as the input data unigene for multivariate COX regression analysis. Thus, the genes that were retained after passing the three checkpoints become components in the prognostic classification model.

### Immune trait analysis

The LM22 file included in the “CIBERSORT” package [28] and ICC expression matrix were read, and the immune cell abundance in each sample was obtained by running CIBERSORT. Groups were grouped based on the risk score calculated by the classification model, and then the difference in the abundance of immune infiltrates between groups was visualized. The expression matrix of ICC was read into R and converted to gct format. After running ESTIMATE [29], stromal score and immune score were obtained, and then the correlation heatmap between ICC and risk score was visualized. The cell population and expression matrix of the ICC were also read by MCP-Counter [30], which was run to obtain the abundance scores of the ten cell species for each sample as the geometric mean of cell type-specific gene expression values. In addition, we obtained immune checkpoint-related genes from previous studies [31], as well as calculated differences in 28 immune cells between patients in different risk groups based on the GSVA package [22].

### Drug sensitivity analysis

According to the Cancer Genome Project (CGP) cell line expression matrix and drug sensitivity information as the training set, “pRRophetic” [32] analyzed the expression matrix of ICC, so as to obtain the IC50 value of the drug response of the sample. The correlation coefficient between drug IC50 values and risk score was calculated by Spearman correlation analysis and visualized as a club-and-stick plot. The significance of the difference of IC50 values between the risk groups was calculated by T test.

### Statistic analysis

All statistics were analyzed in the built-in packages of R software (version 3.6.0). Statistical significance between the two groups was determined by Student’s t-test and wilcoxon rank-sum test. Kaplan–Meier and ROC curves were used to assess the prognosis of the performance of the classification model. Both Sperarman correlation analysis and Pearson correlation analysis were applied in this study. A p value < 0.05 indicated statistical significance.

## Results

### Characteristic annotation and prognostic significance of CSCs in ICC

**Fig. 1:**
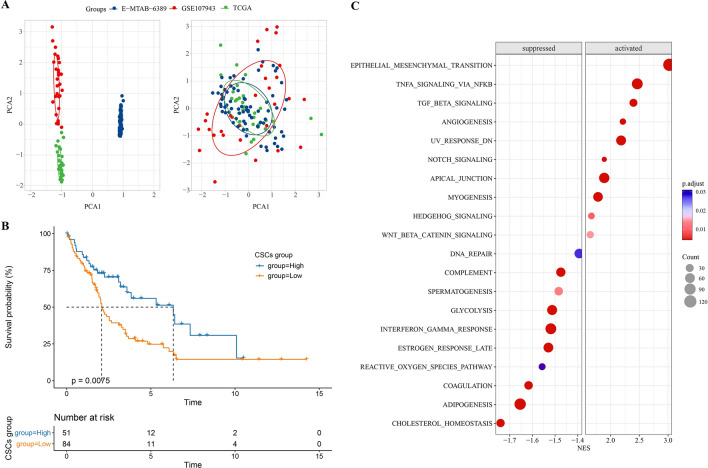
Characteristic annotation and prognostic significance of CSCs in ICC. **A** A 2D PCA distribution plot for batch effects and samples with batch effects removed between the three datasets. **B** Survival differences over time between high and low CSC score categories. **C** NES in high CSC score group compared to low CSC score group. NES > 0 indicated activation, while NES < 0 indicated inhibition

The TCGA-CHOL, GSE107943 and E-MTAB-6389 data sets had batch effects after merging, which were removed by Limma package (Fig. 1A). After calculating the CSCs scores of the sample, the whole cohort was divided into two categories to judge the relationship with ICC prognosis. Samples with low CSC score showed significantly shortened survival time compared to samples with high CSC score (Fig. 1B). To determine the effect of CSCs on cancer-related signaling in ICC, NES of high CSC score group versus low CSC score group was calculated, with NES > 0 indicating activation and NES < 0 indicating inhibition. NFκB and TGFβ signaling were significantly activated in samples with high CSC scores; While cholesterol homeostasis, adipogenesis, reactive oxygen species pathway, glycolysis and immune pathways (complement, interferon gamma response) were significantly suppressed (Fig. 1C). These results support the important role of CSCs in ICC progression and patient prognosis, suggesting that CSCs-based studies provide a new direction for ICC.

### Identification of module associated with CSCs

**Fig. 2:**
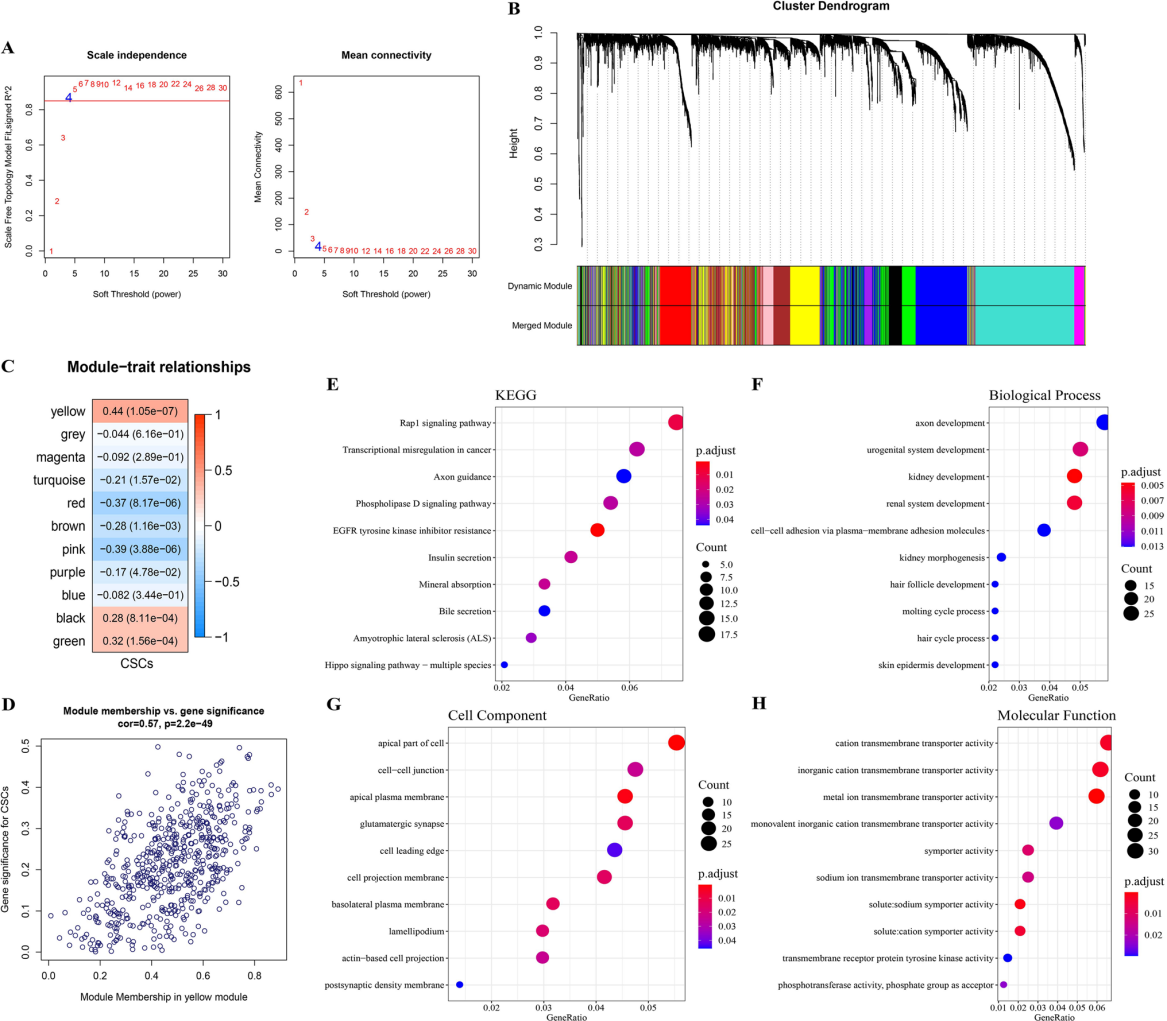
Identification of module associated with CSCs. **A** Scale-free fit exponents and mean connectivity corresponding to different soft threshold powers. **B** Cluster dendrogram of modules. **C** Correlation Heatmap between CSC score and module eigengenes for ICC. **D** The scatter plot illustrates the correlation between gene significance (GS) for CSCs and module membership (MM) in yellow module. **E** KEGG analysis of genes in yellow module. **F**–**H** GO analysis of genes in the yellow module, including biological process (BP), cell component (CC) and molecular function (MF)

When WGCNA is run, selecting the soft threshold is the key to construct the network topology analysis. We chose the first β = 4 with a scale-free topological fit index above 0.85, and the mean connectivity of the genes within the corresponding co-expression module has stabilized, indicating that the network is well connected (Fig. 2A). Modules of ICC were generated by hierarchical clustering. Modules with similar expression patterns were combined according to the similarity of module eigenvalues, and 11 co-expression modules were identified. (Fig. 2B). The heat map generated by the correlation analysis between the module eigenvalues and the CSC score indicated that the yellow module had the most significant correlation with the CSC score (Fig. 2C). There was also a high positive correlation between gene significance (GS) for CSCs and module membership (MM) in yellow module, indicating that genes in yellow module may play a pivotal role in the regulation of stem cell properties of ICC (Fig. 2D). To be specific, genes in yellow module were significantly linked with pathways and biological processes such as Rap1 signaling pathway, transcriptional misregulation in cancer, axon guidance and EGFR tyrosine kinase inhibitor resistance (Fig. 2E–H). Therefore, we will utilize the genes in the yellow module for further analysis.

### Prognostic classification model and its performance

**Fig. 3:**
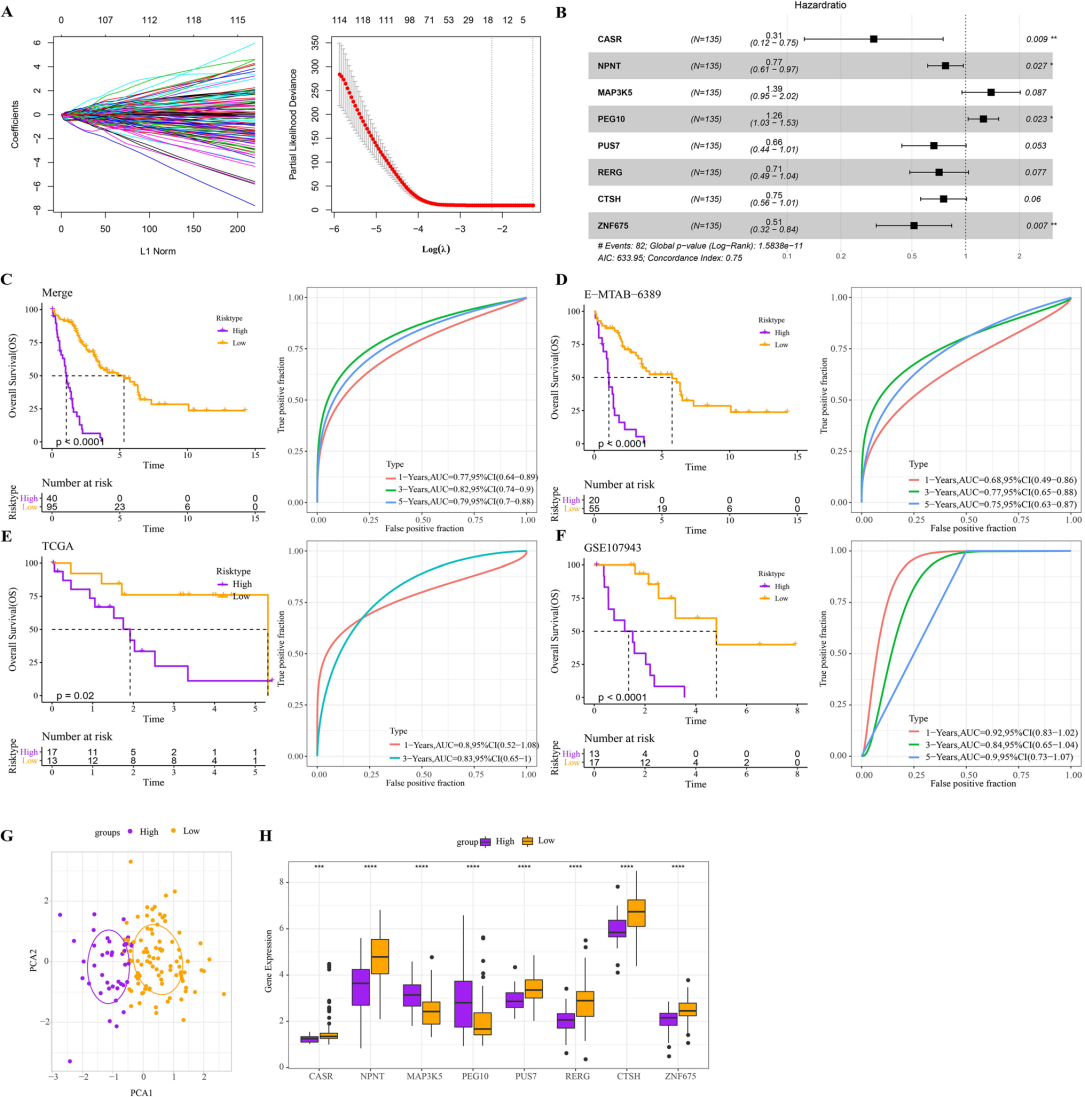
Prognostic classification model and its performance. **A** Dynamic process plot of genes screened by Lasso and errors resulting from tenfold cross-validation. **B** Forest plot for Multivariate Cox regression of genes included in the classification model. **C**–**F** Kaplan–Meier curves and ROC curves of prognostic classification models over time in the **C** combined cohort, **D** E − MTAB − 6389, **E** TCGA-CHOL, and **F** GSE107943 datasets. **G** PCA shows the clustering results of the high-risk and low-risk categories in the combined cohort. **H** The difference in expression of any gene between the two risk categories of the prognostic classification model

The genes in the yellow module were extracted for univariate Cox regression analysis and Lasso regression analysis, and the corresponding upper abscissa value was 18 when the error of tenfold cross validation was the minimum, so 18 genes in the yellow module were candidates for the classification model (Fig. 3A). Genes that were finally included in the classification model were determined by stepwise multivariate regression analysis (Fig. 3B) with the formula: Risk score = (− 1.186*CASR) + (− 0.26*NPNT) + (0.328*MAP3K5) + (0.229*PEG10) + (− 0.409*PUS7) + (− 0.342*RERG) + (− 0.284*CTSH) + (− 0.666*ZNF675). This formula calculated the risk scores of the samples in the pooled cohort and was divided into two risk categories by “survminer”. A steeper decline in survival over time was detected in the high-risk category than in the low-risk category, both in the pooled cohort and in the independent cohort. In the combined cohort, the ROC curves generated according to the prognostic classification model showed that the accuracy of the model in predicting both short-term and long-term outcomes reached the expected high level. In the E-MTAB-6389 cohort, the prognostic classification model had excellent accuracy in predicting 3- and 5-year survival with AUC values of 0.68, 0.77, and 0.75 at 1,3, and 5 years, respectively. And the prognostic classification model was more ideal in predicting prognosis in TCGA-CHOL (AUC values were 0.8 and 0.83 at 1 and 3 years, respectively) and GSE107943 datasets (AUC values were 0.92,0.84 and 0.9 at 1, 3 and 5 years respectively) (Fig. 3C–F). In the results given by PCA, we can also observe good discrimination between the high-risk and low-risk groups (Fig. 3G). When any gene in the prognostic classification model was singled out, significant differential expression was found between the two risk categories. The over-expressed model genes in the high-risk category included MRP3K5 and PEG10, while the remaining six genes were deficient in the high-risk category (Fig. 3H).

### Immune characteristics and response to antineoplastic drugs of prognostic classification model

**Fig. 4:**
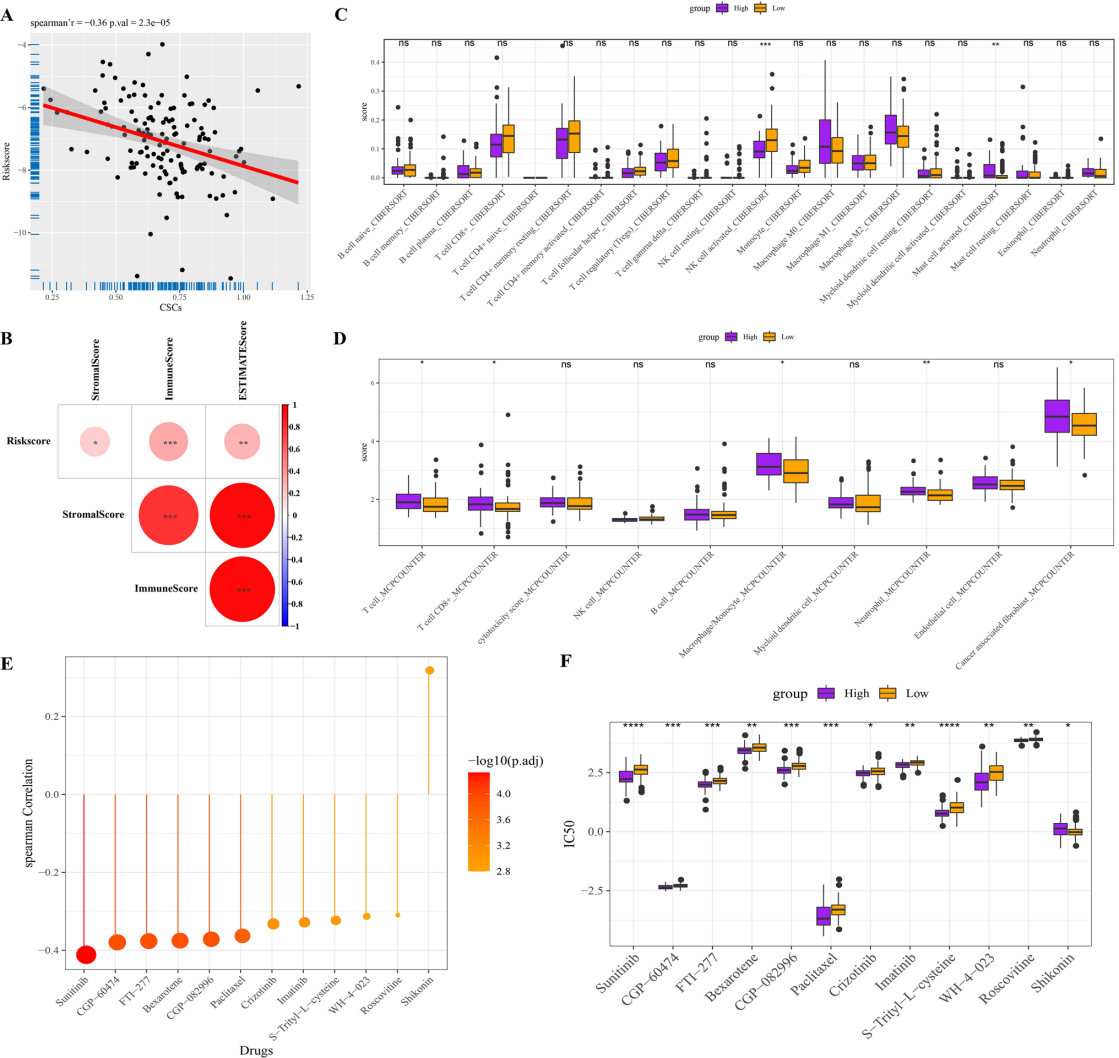
Immune characteristics and response to antineoplastic drugs of prognostic classification model. **A** Scatter plot of Spearman correlation analysis between risk score and CSCs. **B** Heatmap of correlation between risk score and immune score, stomal score and ESTIMATE score. **C**, **D** Abundance of immune infiltrates for the two risk categories, **C** is the result of the CIBERSORT assay and **D** is the result of the MCP-Counter assay. **E** Spearman correlation analysis between risk score and IC50 values of 12 antineoplastic drugs. **F** The IC50 value difference for antineoplastic drugs with a correlation coefficient > 0.3 with risk score between the two risk categories

We analyzed biological traits that might be relevant to prognostic classification models. Risk score was shown to have a significant inverse relationship with CSCs (Fig. 4A). The risk score also showed a significant positive correlation with the macroscopic immune score and stromal score of TME (Fig. 4B). Subsequently, we found that the expression of immune checkpoint-associated genes was also differential among patients in different risk groups. For example, the immune checkpoint activation genes CEACAM1 and TNFSF4 were highly expressed in high-risk patients, whereas the immune checkpoint inhibition genes VTCN1 and SIRPA were significantly highly expressed in low-risk patients (Supplementary Fig. 1A, B).

The detected immune cells significantly associated with the risk score differed depending on the algorithm used. Activated NK cells in the high-risk category were found to be significantly lower than those in the low-risk category only in the results of the CIBERSORT analysis, while activated mast cells showed the opposite situation (Fig. 4C). And it’s very clear from the MCP-Counter results that the absolute abundance of T cell, CD8 T cell, macrophage/monocyte, neutrophil, cancer associated fibroblast in samples from the high-risk category was significantly higher than in samples from the low-risk category (Fig. 4D). In addition, we also assessed the differences in the infiltration of 28 immune cells in patients of different risk groups using the ssGSEA algorithm as well. Similarly, we found that immune cell infiltration of memory B cells, regulatory T cells, T follicular helper cells, neutrophils, and NK T cells was significantly increased in high-risk patients relative to patients in the low-risk group (Supplementary Fig. 1C). A total of 12 antineoplastic drugs corelated with risk score with correlation coefficients |R|> 0.3 were also selected for ICC, among which only the IC50 value of Shikonin was significantly positively correlated with risk score, and rest Sunitinib, CGP − 60474, FTI − 277, Bexarotene, CGP − 082996, Paclitaxel, Crizotinib, Imatinib, S − Trityl − L − cysteine, WH − 4 − 023, Roscovitine were significantly negatively correlated with risk score, respectively (Fig. 4E). Correspondingly, antineoplastic drugs were more suitable for the treatment of high-risk group (Fig. 4F).

### Effect of prognostic classification model on immunotherapy response

**Fig. 5:**
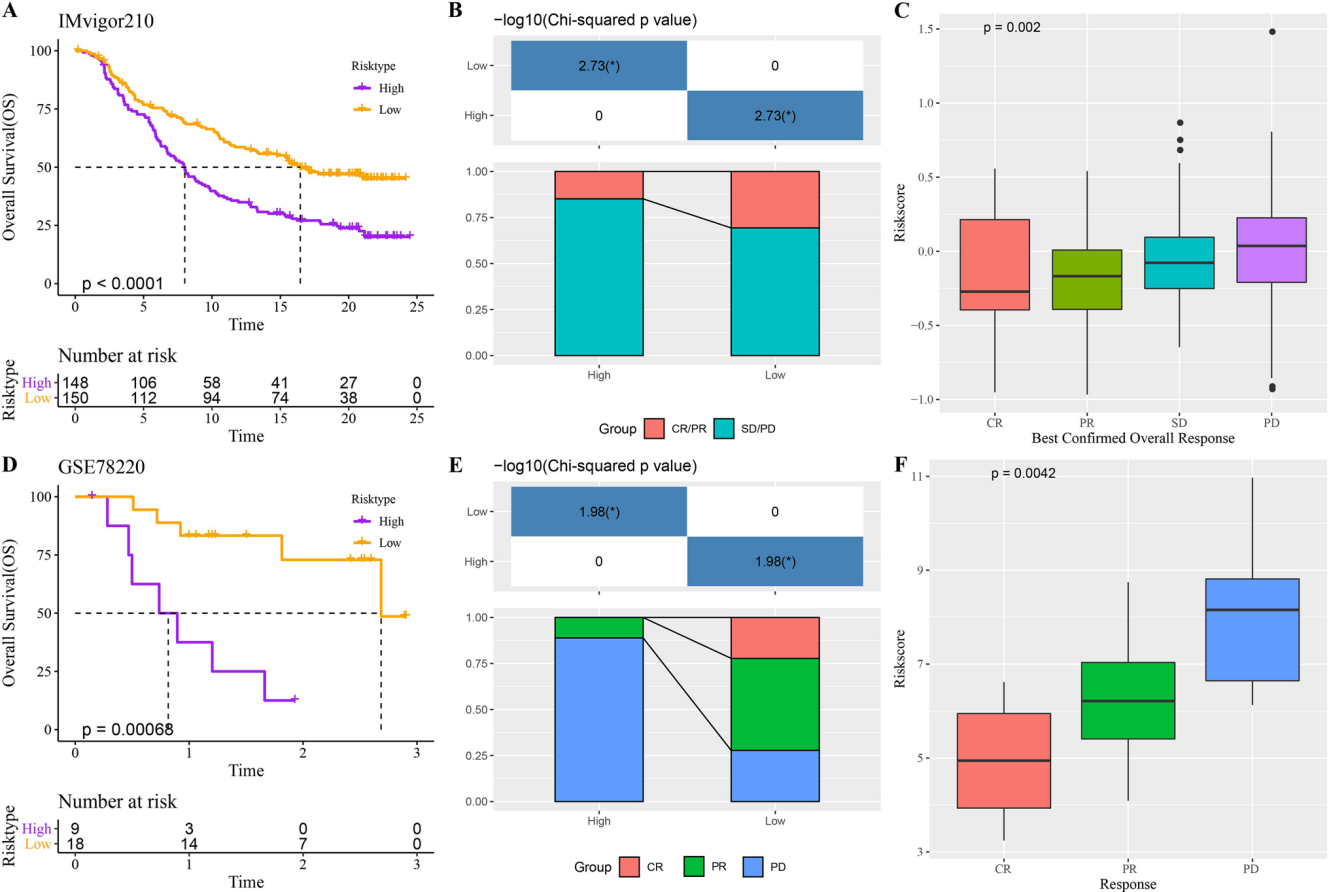
Effect of prognostic classification model on immunotherapy response. **A**, **B** Prediction of prognosis (**A**) and response to immunotherapy (**B**) by the prognostic classification model in the IMvigor210 cohort. **C** Risk scores for samples in the IMvigor210 cohort that showed different responses to immunotherapy. **D**, **E** Prediction results of the prognostic classification model for prognosis (**D**) and immunotherapy response (**E**) in the GSE78220 cohort. **F** Risk scores for samples in the GSE78220 cohort that showed different responses to immunotherapy

The predictive effect of prognostic classification model on prognosis and immunotherapy response was evaluated in two immunotherapy cohorts IMvigor210 and GSE78220. Significant differences in prognosis were detected between high-risk and low-risk categories in both cohorts (Fig. 5A, D). In the IMvigor210 cohort, the proportion of patients with complete response (CR) and partial response (PR) was significantly higher in the low-risk category than in the high-risk category (Fig. 5B). The proportion of samples with CR/PR was significantly higher than that of samples with stable disease (SD) and progressive disease (PD) (Fig. 5C). In the GSE78220 cohort, the proportion of samples exhibiting CR/PR in the low-risk group was also much higher than that in the high-risk group (Fig. 5E). Similarly, the CR samples had significantly higher risk scores than the PR samples, and the PR samples had significantly higher risk scores than the PD samples (Fig. 5F). These results illustrate the significant discriminatory power of risk classification models in predicting the corresponding as well as overall survival of ICC patients to immunotherapy. Specifically, patients in the low-risk group had better survival and response rates to immunotherapy than patients in the high-risk group.

### Underlying biological mechanism of prognostic classification model regulating ICC

**Fig. 6:**
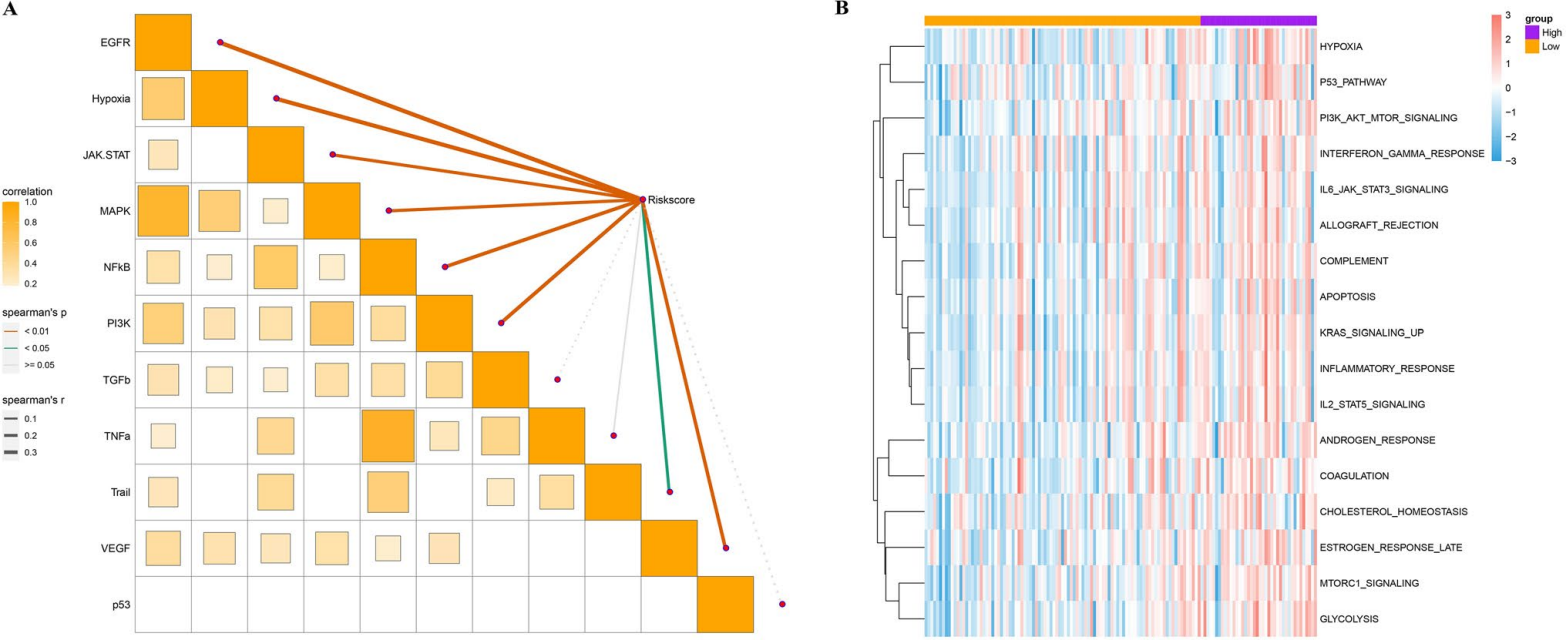
Underlying biological mechanism of prognostic classification model regulating ICC. **A** Spearman correlation analysis between enrichment scores of signaling pathways regulated by signature cancers and risk scores. **B** Heatmap of enrichment of hallmark pathways in high-risk and low-risk categories

The enrichment scores of signature cancer regulatory signaling pathways in each sample were calculated using the R package progeny, followed by Spearman correlation analysis with the risk score. Risk score showed significant positive correlation with EGFR signaling, hypoxia, JAK-STAT signaling, MAPK signaling, NFκB signaling, PI3K signaling, Trail signaling, VEGF signaling (Fig. 6A). hypoxia, PI3K-AKT-mTOR signaling, KRAS signaling up and numerous immunomodulatory pathways were extremely active in the high-risk category, whereas these pathways were inactive in the low-risk category (Fig. 6B). Abnormal activation of these pathways may be a potential driver of adverse prognosis in high-risk category.

## Discussion

Accumulating evidence suggests that the tumor tissue of ICC is enriched in CSC populations and implies that ICC is a stem cell-based disease [33]. However, information on CSC biology in ICC is very limited due to the lack of an ICC-CSC model to characterize [34]. In this study, we first explored the prognostic significance of CSCs for ICC and the potential regulatory pathway of its tumorigenesis. We found that a high CSC score was also beneficial for the survival of ICC patients, accompanied by significant activation of epithelial-mesenchymal transition, angiogenesis, Notch signaling and TGFβ signaling. EMT has been found to induce stem cell properties in the context of cancer [35, 36]. Angiogenesis, Notch pathway, and TGF-β signaling are also essential factors and molecular signals for maintaining CSC characteristics [37, 38]. CSC may be able to evade the immune system by altering its immunogenicity, thereby avoiding rejection mediated by the tumor immune system [39]. Here, we found that the immune pathways complement and interferon gamma response were significantly suppressed in patients exhibiting high CSC scores, which may be able to provide potential support for this argument.

CSC markers and CSC-specific transcriptional signatures have been reported to be functionally associated with aggressive behavior and highly predict overall survival in patients with solid cancer [40]. Although a set of markers is thought to recognize CSC pool in various cancers, further research is needed to identify specific CSC markers for cancer types [41]. Notably, it may be difficult to eliminate CSCs by targeting a single molecular marker or signaling pathway due to the crosstalk between CSCs and different cells in the tumor microenvironment (TME) [42]. We observed an increased infiltration of immune cells such as neutrophils and mast cells in the high-risk group of patients. Watanabe et al. then retrospectively evaluated 44 patients with mass-forming ICC and found that the degree of neutrophil infiltration at the tumor site was significantly and positively correlated with the patients' poorer recurrence-free survival [43]. In addition, mast cells have been reported to promote tumor development by releasing angiogenic and lymphangiogenic factors, as well as molecules such as histamine [44]. These findings suggest that CSC significantly alter the tumor microenvironment through interactions with immune cells, which are inextricably linked to the prognosis of patients with ICC and the promotion of cancer progression. In addition, in this study, the focus was lay on the combination of multiple molecular marker parameters, as the analysis of multivariate biomarkers not only assesses tumor recurrence risk in a post-treatment setting, but also provides information about therapeutically addresable targets in tumor tissue [45]. We constructed a prognostic classification model based on eight CSC markers, and the risk score obtained by this model was significantly negatively correlated with CSCs, which not only effectively predicted the prognosis of ICC patients, but also guided the treatment decision-making, specifically, in identifying ICC patients suitable for chemotherapy and immune checkpoint inhibitor treatment. Furthermore, important insights into the underlying molecular mechanisms driving tumor progression can be gained through prognostic modeling.

CaSR and MAP3K5 in the prognostic classification model have been reported to be involved in the molecular regulation of ICC. CaSR is upregulated in ICC tumor specimens and cell lines, acting as an oncogene to drive ICC progression and poor prognosis [46]. MAP3K5 is a participant in the MAPK signaling cascade and is normally inactivated by thioredoxin binding, whose activity was activated by Chaetocin in ICC cells, and then directly activated the downstream JNK pathway to induce cell apoptosis [47]. Other CSC markers in prognostic classification models have also been reported in diversified solid tumors. NPN expression is associated with the metastatic propensity of breast cancer cells, promoting metastasis through its integrin binding motif, and forced expression in breast tumor cells also promotes their colonization in the lung [48]. An analysis of the Cancer Genome Atlas has shown that PEG10 is associated with poor overall survival in colorectal cancer and functionally contributes to the aggressive progression of colorectal cancer [49]. RERG is frequently silenced by promoter CpG methylation in nasopharyngeal carcinoma and inhibits the metastatic process and angiogenesis of NPC by mediating ERK/NF-κB signaling pathway [50]. CTSH mediates talin processing and promotes prostate cancer migration by regulating integrin activation and adhesion strength [51]. The above literature suggests that genes in prognostic classification models play an important role in the physiology of solid tumors. Collectively, these genes regulate the development of ICC and influence cancer progression and prognosis through complex mechanisms. Therefore, they may serve as risk factors for assessing the clinical prognosis of ICC patients.

There were several limitations that should be stated in the present study. First, this study relies heavily on information from public databases, which can suffer from limited sample size and data bias. In the future, patients of different races, genders, and ages will be included to enable a more comprehensive understanding of the association between CSC characteristics and TME. In addition, our conclusions were mainly based on bioinformatics analysis and lacked practical experimental validation, which prompted us to add multiple cellular experiments, animal models, and clinical samples to validate these findings in future studies. Finally, the TME is complex, encompassing these multiple types of cellular and molecular interactions, which needs to be realized by incorporating cutting-edge technologies such as single-cell sequencing and spatial transcriptomics in further studies to reveal a higher-resolution picture of the TME.

## Conclusion

Taken together, the results of the analysis presented in this study reveal the regulatory role of CSC-mediated EMT, angiogenesis, and immune regulation of biological processes in ICC. Our work also demonstrated a favorable prognostic classification model for ICC, which may be a potentially valuable tool to guide treatment decisions and improve patient outcomes.

### Supplementary Information

Supplementary Material 1.

## Data Availability

The dataset analyzed in this study is available in [GSE107943] at [https://www.ncbi.nlm.nih.gov/geo/query/acc.cgi?acc=GSE107943] and [GSE78220] at [https://www.ncbi.nlm.nih.gov/geo/query/acc.cgi?acc=GSE78220].

## Abbreviations

ICC: Intrahepatic cholangiocarcinoma
CSC: Cancer stem cell
EMT: Epithelial-mesenchymal transition
LASSO: Least absolute shrinkage and selection operator
ROC: Receiver Operating Characteristic
TCGA: The Cancer Genome Atlas database
GEO: Gene Expression Omnibus
WGCNA: Weighted gene co-expression network analysis
GSEA: Gene set enrichment analysis
NES: Normalized enrichment score
CGP: Cancer Genome Project
GS: Gene significance
MM: Module membership
CR: Complete response
PR: Partial response
SD: Stable disease
PD: Progressive disease
TME: Tumor microenvironment
