# Supplementary figures and images for: The potential of cancer stem cells for personalized risk assessment and therapeutic intervention in individuals with intrahepatic cholangiocarcinoma
Source: Discov Oncol. 2024 Jul 24;15:306. doi: 10.1007/s12672-024-01179-7 (PMC11269542; doi:10.1007/s12672-024-01179-7)

**A****Activate**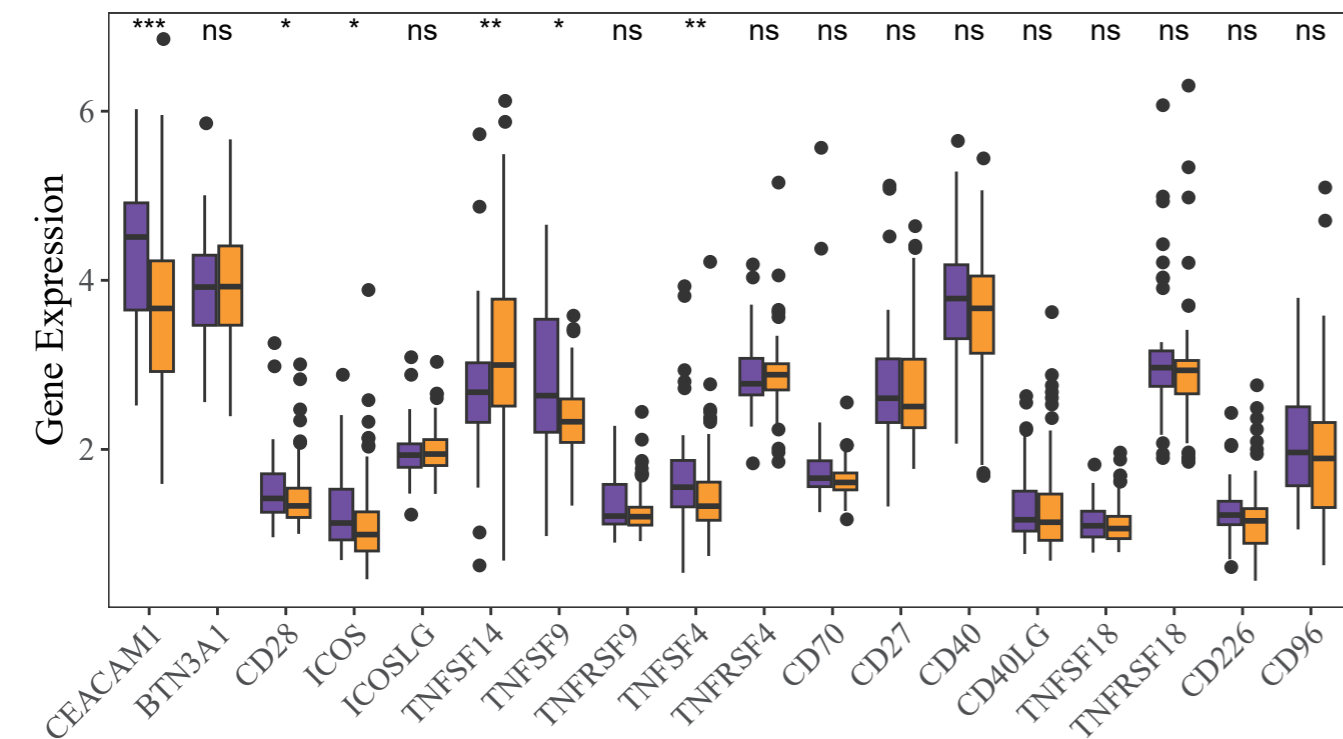**B****Inhibit**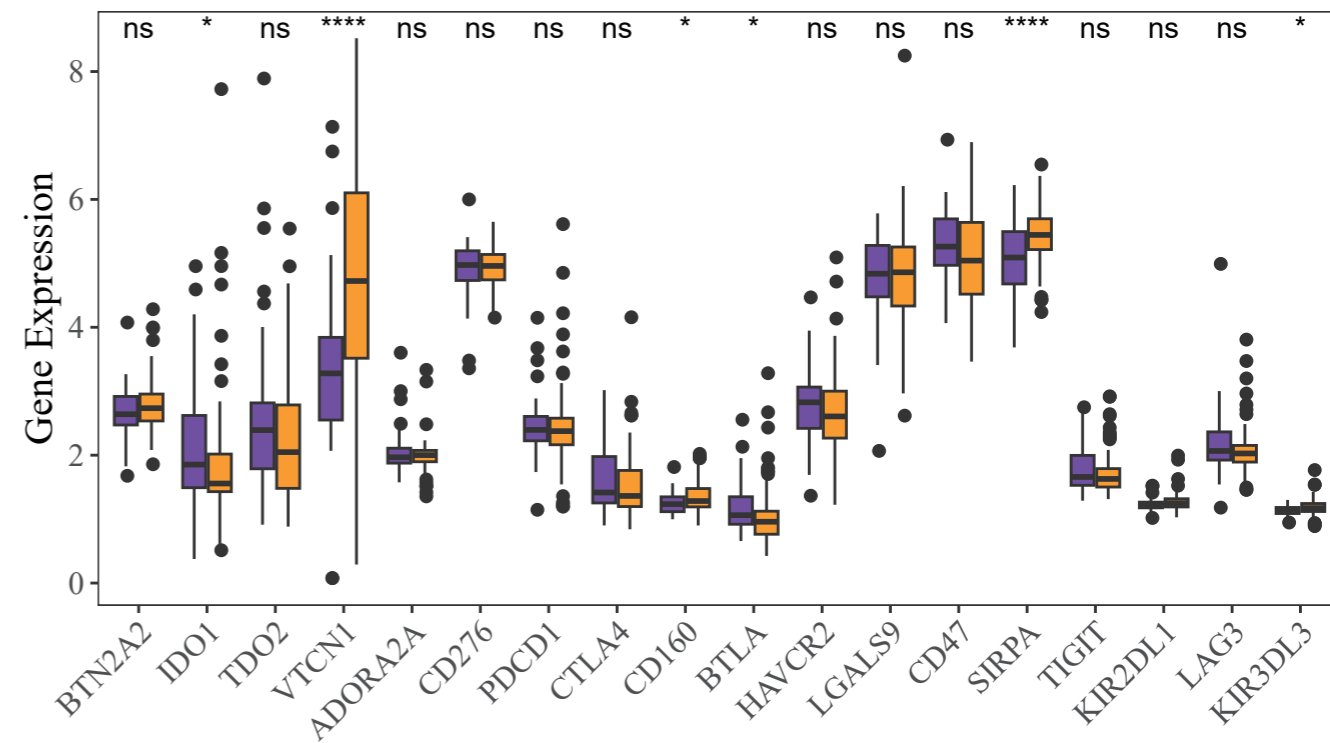**C**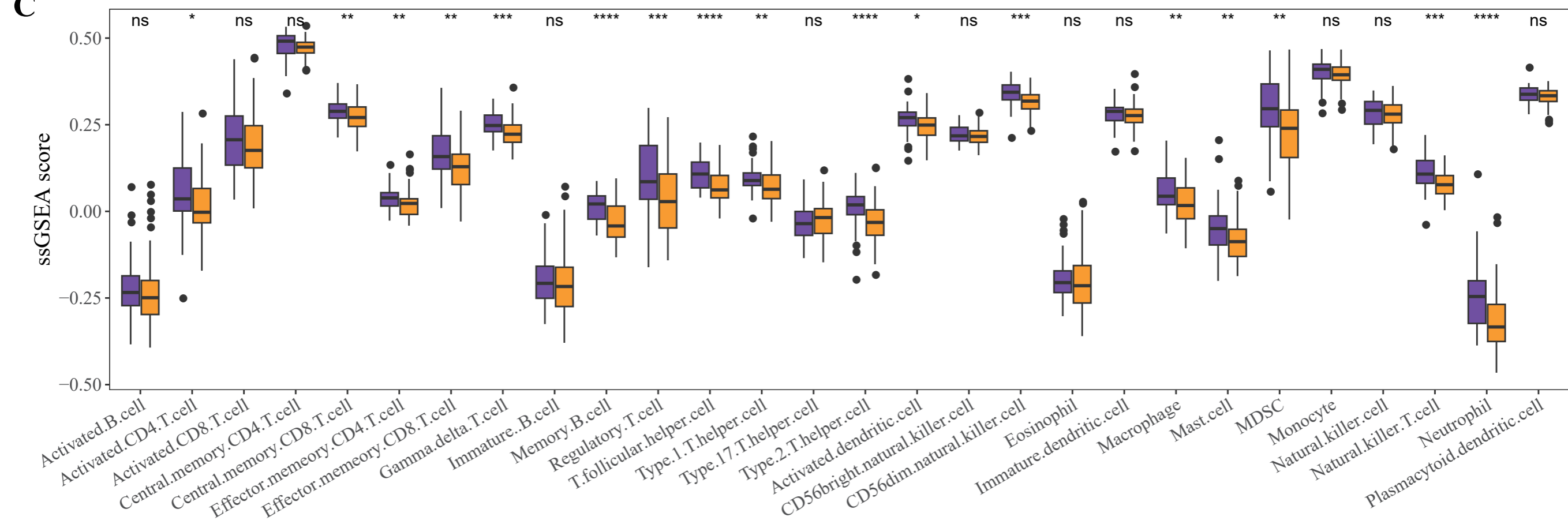

Supplement: Supplementary file 1 — Supplementary Material 1. [file 12672_2024_1179_MOESM1_ESM.pdf]
